# THE ATTITUDE OF MEDICAL PRACTICES TOWARD LGBTQ OLDER ADULTS BEFORE AND AFTER INTERVENTION

**DOI:** 10.1093/geroni/igz038.1822

**Published:** 2019-11-08

**Authors:** Mackenzi Kim, Lynn M Wilson, Nyann Biery, Brenda Frutos

**Affiliations:** Lehigh Valley Health Network, Allentown, Pennsylvania, United States

## Abstract

Individuals who identify as lesbian, gay, bisexual, transgender or other non-heterosexual or binary gender identifiers (LGBTQ) face tremendous obstacles in search of quality healthcare. Older LGBTQ adults face these obstacles in the setting of more complex health problems with few social services and support. Negative treatment from healthcare professionals has proven to be one of the most pervasive barriers to care faced by older LGBTQ adults. Sensitization training with the film, Gen Silent, is one way knowledge gaps and biases of healthcare professionals has been addressed. By utilizing the survey previously validated by Porter et al., health professionals’ knowledge, perceptions, and attitudes toward LGBTQ older adults before and after viewing Gen Silent were assessed in Lehigh Valley Health Network (LVHN)-affiliated primary care practices. The principle outcome of this study was a statistically significant change in responses. Primary care practices were recruited for 45-minute sessions that included the showing of an educational, abbreviated version of Gen Silent to available staff. It was preceded by administration of a pretest survey and followed by a posttest survey and discussion. A paired t-test was conducted to determine significance of differences between pre- and posttest responses. Seventeen individuals (N=17) viewed the film and finished pre- and posttest surveys. Nearly all questions exhibited changes between pre- and posttests. Significantly, respondents indicated increased awareness of additional barriers to care faced by LGBTQ older adults compared to heterosexual peers. While limited, these results indicate that primary care professionals would benefit from training specific to the aging LGBT population.

